# Clustering of asthma and related comorbidities and their association with maternal health during pregnancy: evidence from an Australian birth cohort

**DOI:** 10.1186/s12889-021-11997-x

**Published:** 2021-10-27

**Authors:** Kabir Ahmad, Enamul Kabir, Gail M. Ormsby, Rasheda Khanam

**Affiliations:** 1 School of Business, University of Southern Queensland, Toowoomba, QLD 4350 Australia; 2 Centre for Health Research, University of Southern Queensland, Toowoomba, QLD 4350 Australia; 3 School of Sciences, University of Southern Queensland, Toowoomba, QLD 4350 Australia; 4 Independent Researcher, School of Education, Faculty of Business, Education, Law and Arts, University of Southern Queensland, Toowoomba, QLD 4350 Australia

**Keywords:** Latent class analysis, Cluster analysis, Asthma, Asthma related comorbidities, Paediatric quality of life, General well-being, Spirometry

## Abstract

**Background:**

The population-based classification of asthma severity is varied and needs further classification. This study identified clusters of asthma and related comorbidities of Australian children aged 12–13 years; determined health outcome differences among clusters; and investigated the associations between maternal asthma and other health conditions during pregnancy and the children’s clustered groups.

**Methods:**

Participants were 1777 children in the birth cohort of the Longitudinal Study of Australian Children (LSAC) who participated in the Health CheckPoint survey and the LSAC 7th Wave. A latent class analysis (LCA) was conducted to identify clusters of children afflicted with eight diseases, such as asthma (ever diagnosed or current), wheezing, eczema, sleep problem/snoring/breathing problem, general health status, having any health condition and food allergy. Multinomial logistic regression was used to investigate the association between maternal asthma or other health conditions and LCA clusters.

**Results:**

The study identified four clusters: (i) had asthma – currently healthy (11.0%), (ii) never asthmatic & healthy (64.9%), (iii) early-onset asthmatic or allergic (10.7%), and (iv) asthmatic unhealthy (13.4%). The asthmatic unhealthy cluster was in poor health in terms of health-related quality of life, general wellbeing and lung functions compared to other clusters. Children whose mothers had asthma during pregnancy were 3.31 times (OR 3.31, 95% CI: 2.06–5.30) more likely to be in the *asthmatic unhealthy* cluster than children whose mothers were non-asthmatic during pregnancy.

**Conclusion:**

Using LCA analysis, this study improved a classification strategy for children with asthma and related morbidities to identify the most vulnerable groups within a population-based sample.

## Background

Asthma, a chronic respiratory disease, poses a significant global health burden, particularly in developed countries [1]. This heterogeneous respiratory disorder is comprised of differing characteristics and phenotypes [1]. Globally, there were more than 262 million people affected by asthma in 2019 and caused 461,000 deaths [2]. The 2020 Australian health study revealed that around 11% of the Australian population (2.7 million) had asthma in 2017–18; during that time there were 38,792 hospitalizations for asthma, 80% of which were preventable [3]. The 2018 Australian health report mentioned that, as per Australian Burden of Disease Study, asthma is the leading cause of burden among children aged 5–14 years [4]. A longitudinal study from the birth cohort of 2004, conducted in 2015, found that 16.9% of Australian children experienced wheezing or asthma within the first 3 years of life [5]. Asthma is more prevalent chronic disease among children and young adults than adults, particularly because of its early onset [6] and diverse symptoms accompanied by other comorbidities - wheezing, atopic allergy, food allergy or poor health [7].

Current descriptions of asthma phenotypes and its classifications have been identified but have not considered several other domains of comorbidities, such as eczema, snoring/breathing problems or food allergies, related with asthma [8, 9] Inclusion of these related diseases with asthma and the use of a classification system may provide a framework to identify distinct asthma phenotypes and a better understanding of its aetiology.

Currently, the literature describes diverse classifications of the cluster analysis of asthma phenotypes. An UK study identified the clusters according to varying combinations of wheezing disorders, atopic allergies, and impaired lung functions with high or low severity of asthma [8]. In the USA, Moore et al. (2010) identified clusters within the Severe Asthma Research Program cohort based on distinct clinical phenotypes using unsupervised hierarchical cluster analysis. However, they also acknowledged the need for an improved classification of asthma morbidities [10]. Similarly, in an European study, Siroux et al. (2011) proposed latent class analysis (LCA) to improve asthma morbidities classification utilizing multiple aspects of the disease in adults who participated in an epidemiological study [11]. The findings revealed different homogeneous groups with severe and mild asthma whose different phenotypes, allowed them to differentiate the quality of life and associated risk factors [11]. A New Zealand study (Wellington Respiratory Survey) assessed clinical airway diseases and found varying aspects of asthma and related comorbidities in five distinct clusters of the population [12]. Many of these asthma clustering studies were conducted outside of Australia, and few studies have used a model-based cluster analysis of asthma and related comorbidities using a nationally representative sample.

Epidemiological studies suggest that certain health conditions such as having asthma or being overweight during pregnancy, are associated with childhood asthma [13–17]. However, many LCAs or cluster analyses on asthma comorbidities in children lack an investigation of the foetal origins of the children’s cluster memberships [8, 10]. Furthermore, adolescence is a crucial phase in the life cycle [18] and a critical entry point for young people approaching adulthood [19].

Thus, the primary purpose of this study was to identify clusters of asthma and related comorbidities (wheezing, eczema and others) among Australian adolescent children (12/13 years of age in 2016) from the birth cohort of LSAC study recruited in 2004–2005. Secondly, the objective was to identify each cluster’s characteristics and determine their differences as measured by spirometry tests, paediatric quality of life (PedsQL), and general wellbeing, in order to identify the most vulnerable cluster. Lastly, the study aimed to investigate potential associations between maternal health status during pregnancy and the health outcomes among the clusters of adolescents.

## Methods

### Setting and data

**Fig. 1:**
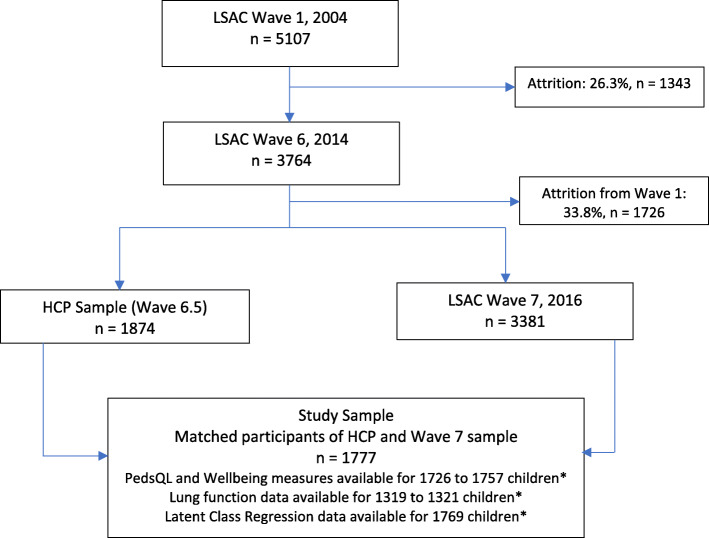
Participant diagram of the study. Notes: *For the comparisons on the health outcome variables of the study sample, observations with missing values were excluded; n = number of families/Children. Abbreviations: HCP, Child Health CheckPoint; LSAC, Longitudinal Survey of Australian Children; PedsQL, Paediatric Quality of Life

The study participants were 1777 Australian children aged 12–13 years, who participated in both the Health CheckPoint (HCP) survey and the 7th Wave of LSAC, conducted between 2015 and 2016. The LSAC is a prospective, nationally representative longitudinal household survey gathering data on a wide range of factors that influence child development. The LSAC commenced in 2004 and collects data every 2 years. The HCP survey was a special health assessment offered to the children in LSAC between Waves 6 and 7, in 2015. It assessed several health measurements and bio-specimens, including respiratory measurements. Details of the study designs and recruitment processes for the LSAC and HCP surveys are provided elsewhere [20–22]. This study performed the Latent Class Analysis (LCA) on the selected 1777 children to identify clusters of children afflicted with asthma and related comorbidities. The PedsQL and wellbeing scores were available for 1726–1757 children. The four lung function measures were available for 1319–1321 children, excluding the respective measures’ missing values (Fig. 1). Comparison of the clusters’ health outcomes were performed based on the available children’s data of the respective measures.

### Latent class analysis variables

The LCA was conducted using asthma and other diseases or symptoms linked with asthma taken from the 7th Wave of LSAC survey. The morbidity variables were asthma (ever diagnosed or current), wheezing, eczema, sleep problem/snoring/breathing problem, general health status, having any health condition and food allergy.

### Health outcome variables

Three groups of health outcome indicators related to asthma and its comorbidities were used in this study: health-related quality of life, general wellbeing, and lung function.

#### Health-Related Quality of Life

PedsQL 4.0 Generic Core Scales, an integral part of LSAC and HCP surveys, are reliable and responsive measures of the health outcomes of both healthy children and children suffering from asthma-related comorbidities [23]. Each child in the study completed a health-related, 23-item questionnaire comprising the following subscales: (i) general health subscale, (ii) general wellbeing subscale, (iii) physical functioning subscale, (iv) emotional functioning subscale, (v) school functioning subscale, and (vi) social functioning subscale [23]. The summary scores of the physical and psychosocial health scales and the total score were calculated from these subscales; a higher value indicates better health. To calculate the scale scores, the mean was computed as the sum of the items over the number of items answered. Details of the procedure are described elsewhere [22, 23].

#### General wellbeing

These wellbeing variables were generated by taking the participants’ responses to a six-item questionnaire, taken from two psychometric subscales used in the International Survey of Children’s Well-being (ISCW). These questions were designed to measure children’s satisfaction with their life as a whole and their satisfaction relating to their family life, friends, school experience, where they live, and their own body, measured on a scale from 0 (not satisfied at all) to 10 (fully satisfied). Then, the five-item Brief Multi-Dimensional Students’ Life Satisfaction Scale (BMSLSS) and single-item Overall Life Satisfaction scale (OLS) were created after converting the total scores into a 100 scale. Details of the process are described in Seligson et al. and health checkpoint data user guide [24, 25].

#### Lung function measures

Spirometry tests to measure the lung function of the children were conducted during the HCP survey. Details of the procedures and steps of these measurements are described by Welsh et al. [26]. The pre-bronchodilator spirometry data were converted to z-scores using the Global Lung Initiative’s 2012 reference eq. [25]. This study included the following lung function measures of the children aged 11–12 years from the HCP survey: forced expiratory volume in 1 s (FEV_1_), forced vital capacity (FVC), FEV_1_/FVC ratio, and mid-expiratory flow (MEF) - which is forced expiratory flow between 25 and 75% of FVC (FEF 25–75%), and their z-scores. These variables were used to compare lung function variations among the clusters, as previous studies have shown that lung function varies with asthma-related morbidities [27].

### Variables of regression analysis

In the regression analysis, this study used maternal health conditions during pregnancy: (i) asthma, (ii) smoking, (iii) obesity status, (iv) having any medical conditions, and two birth related variables: (i) gestational age, and (ii) birth weight of children as the independent variables. The children’s cluster is the dependent variable that has four categories, namely, had asthma- currently healthy, never asthmatic & healthy, early-onset- asthma/allergic, and asthmatic unhealthy. These four categories were developed based on asthma and related comorbidities of children using latent class clustering procedure. The mothers’ socio-economic status and child-related other variables are used as control variables to adjust the regression model.

### Statistical analysis

An LCA was performed to classify 1777 children according to the incidence of asthma and other comorbidities. The analysis aimed to identify groups (classes) of ‘similar’ children using a model-based approach considering the distribution of these comorbidities. The LCA classified the children according to the probabilities of the observed values of all the variables listed in Table 1 for each of the children. We used STATA (version 15.0) to run intercept-only models and to fit the logistic regression models for all the selected cluster variables.

**Table 1:** Goodness of fit statistics for cluster models

Model	LL	BIC	AIC	Npar	L^2^	% reduction in L^2^	residual df	*p*-value
One class (H_0_)	− 4698.805	9464.953	9415.609	9	1330.987		502	0.000
Two class	− 4175.756	8493.682	8389.511	19	284.889	78.6	492	1.000
Three class	− 4154.712	8511.456	8363.423	27	242.802	81.8	484	1.000
Four class	− 4140.697	8550.771	8353.394	36	214.772	83.9	475	1.000
Five class	−4140.679	8625.561	8373.358	46	214.736	83.9	465	1.000
Six class	− 4131.795	8637.725	8363.591	50	196.969	85.2	461	1.000
Seven class	− 4135.595	8660.289	8375.189	52	204.568	84.6	459	1.000

*Abbreviations*: *AIC* Akaike’s Information Criterion, *BIC* Bayesian Information Criterion, *df* degrees of freedom, *Npar* Number of parameters, *LL* log-likelihood, *L*^2^ likelihood-ratio

The goal of LCA was to select a final LCA model that maximized the log-likelihood and minimized the Bayesian Information Criterion (BIC), Akaike’s Information Criterion (AIC) and the likelihood ratio function *L*^*2*^ (deviance statistics). During the analyses, we estimated the model for one to seven latent clusters to obtain the best classification. For each number of clusters, the model was repeated 100 times so that the parameter estimates corresponding to the model could produce the greatest log-likelihood. Sensitivity of clusters/groups was also tested by observing changes of pattern of clusters due to inclusion/exclusion of related variables from the analysis. The optimal number of clusters was determined based on a combination of the log-likelihood, BIC, AIC and the likelihood function (L^2^, the likelihood-ratio/deviance statistics) for achieving the optimal model.

The information criteria values, shown in Table 1, suggested a four-cluster solution based on Akaike’s Information Criterion (AIC) and a two-cluster model based on Bayes’ Information Criterion (BIC). However, 83.9% reduction in L^2^ from one class (H_0_) to four class suggests that four-cluster model is beneficial. In the five or six cluster models, L^2^ reduced by only a further 1.3% or less, hence not so beneficial. On the basis of these results, and the characteristics and size of the clusters, the four-cluster solution was selected as optimal.

Frequency analysis was used to describe the characteristics of children included and excluded from the study. Furthermore, after defining the clusters, cluster-based mean comparison analyses and significance tests of the PedsQL scores, wellbeing scores and spirometry measures were performed. All these statistical measures were weighted to represent the population of Australian children. Subsequently, multinomial logistic regression analysis was conducted to investigate the associations between maternal health-related risk factors and the cluster groups. The regression model was adjusted with control variables and the *never asthmatic & healthy* cluster was considered the reference cluster.

## Results

### Sample characteristics

The percentage distributions of the basic characteristics of the included and excluded LSAC participants of this study in the baseline wave are shown in Table 2. The excluded children were those who could not participate in the 7th wave and in the HCP survey. Among the included children, 50.9% were male, 5.8% weighed less than 2500 g at birth, 62.1% had a normal birth, 8.1% were not immunized completely and 43.9% were not breastfed until 6 months of age. Over one-fourth of the children (28.7%) had been diagnosed with asthma during their lives, and 13.4% were currently suffering from asthma and taking medication. Around one in every ten children had ongoing eczema or wheezing. Only 5.4% mentioned sleeping problems due to snoring or breathing problems.

**Table 2:** Baseline characteristics of the excluded and included children

Characteristics		Children excluded ***n*** = 3330 n (%)	Children included ***n*** = 1777 n (%)
Sex of the study child (1 = male)		1706 (51.2)	904 (50.9)
Birth Weight < 2500 g (yes = 1)		185 (5.6)	103 (5.8)
Gestational age < 37 weeks (yes = 1)		231 (6.9)	119 (6.7)
Breastfed less than 6 months (yes = 1)		1394 (41.9)	779 (43.9)
Type of birth – Normal (Yes = 1)		2132 (64.0)	1103 (62.1)
Type of birth – Caesarean (Yes = 1)		977 (29.3)	542 (30.5)
Immunization not completed (yes = 1)		330 (9.9)	144 (8.1)
Is English spoken at home? (yes = 1)		2845 (85.5)	1612 (90.8)
Is the child Indigenous? (yes = 1)		209 (6.3)	38 (2.1)
Educational Qualification of Mother:			
Year 12 or equivalent (Yes = 1)		1667 (50.1)	1248 (70.2)
University education	Graduate/diploma	904 (27.2)	748 (42.1)
	Post-graduate	161 (4.8)	167 (9.4)
Remoteness of Area	Metropolitan cities	2244 (67.4)	1298 (73.1)
	Inner cities	620 (18.6)	290 (16.3)
	Outer region/ Remote areas	466 (14.0)	189 (10.6)

### Latent class identification

In the latent class cluster analyses, we found that the optimal solution was four clusters (AIC value: 8353.394, BIC value: 8550.771, log-likelihood ratio: 214.772). These clusters were identified as follows: (i) had asthma – currently healthy, (ii) never asthmatic & healthy, (iii) early-onset asthmatic or allergic, and (iv) asthmatic unhealthy. Table 3 shows the prevalence of comorbidities for all the study children and by cluster. Table 4 shows the mean comparisons of the quality of life, wellbeing, and the lung functions among children by cluster.

**Table 3:** Prevalence of asthma and related morbidities by clusters (*n* = 1777)

Asthma and related morbidity criteria of the study child		All Sample (*n* = 1777)	Cluster 1 (***n*** = 195, 11.0%)	Cluster 2 (***n*** = 1154, 64.9%)	Cluster 3 (***n*** = 190, 10.7%)	Cluster 4 (***n*** = 238, 13.4%)
		n (%)	Had asthma- currently healthy n (%)	Never asthmatic & healthy n (%)	Early-onset- asthma/allergic n (%)	Asthmatic unhealthy n (%)
Ever diagnosed with asthma	Yes	510 (28.7)	195 (100)	0 (100)	77 (40.5)	238 (100)
Currently suffering from Asthma	Yes	238 (13.4)	0 (100)	0 (100)	0 (100)	238 (100)
Has illness with wheezing	Yes	155 (8.7)	0 (100)	33 (2.9)	30 (15.8)	92 (38.7)
Has ongoing eczema	Yes	169 (9.5)	16 (8.2)	56 (4.9)	42 (22.1)	55 (23.1)
Diagnosed with sleep problem related with snoring/breathing problem	Yes	96 (5.4)	0 (100)	30 (2.6)	40 (21.1)	26 (10.9)
Reported as not having excellent/very good Health	Yes	198 (11.1)	0 (100)	82 (7.1)	66 (34.7)	50 (21.0)
Has reported at least one health condition	Yes	68 (3.8)	0 (100)	23 (2.0)	25 (13.2)	20 (8.4)
Has food allergy	Yes	155 (8.7)	0 (100)	0 (100)	114 (60.0)	41 (17.2)

**Table 4:** Average scores of PedsQL, well-being and lung function measures by the cluster group of the children

PedsQL, Well-being and Lung Function Measures	Total Sample		Cluster 1		Cluster 2		Cluster 3	Cluster 4			*p*-value
	n	Mean (SD)	n	Mean (SD)	n	Mean (SD)	n	Mean (SD)	n	Mean (SD)	
**A. Paediatric Quality of Life Inventory**											
Inventory physical health summary	1750	83.1 (13.6)	192	84.5 (12.3)	1141	84.0 (13.0)	181	81.5 (13.6)	236	79.4 (16.4)	**< 0.001**
Inventory psychosocial health summary	1750	75.8 (14.6)	191	77.4 (14.7)	1141	76.7 (14.0)	182	72.1 (15.2)	236	73.1 (15.9)	**< 0.001**
Inventory total score	1751	78.4 (13.0)	192	79.9 (13.1)	1141	79.3 (12.6)	182	75.4 (13.3)	236	75.3 (14.2)	**< 0.001**
**B. International Survey of Children’s Well-being**											
Brief Multi-dimensional Students’ Life Satisfaction Scale	1750	82.8 (13.8)	190	85.0 (13.4)	1143	83.0 (13.7)	182	81.0 (14.2)	235	81.5 (14.5)	**0.014**
Overall Life Satisfaction	1745	80.6 (18.5)	190	84.3 (17.7)	1137	81.0 (18.1)	183	78.7 (17.7)	235	77.5 (21.2)	**< 0.001**
**C. Lung Function Measures**											
**Raw**											
FEV_1_ (L)	1321	2.54 (0.42)	147	2.54 (0.40)	858	2.57 (0.41)	134	2.51 (0.42)	182	2.46 (0.42)	**0.016**
FVC (L)	1321	2.99 (0.51)	147	3.03 (0.51)	858	2.99 (0.51)	134	2.95 (0.51)	182	2.96 (0.50)	0.493
FEV_1_/FVC ratio	1319	0.88 (0.02)	147	0.87 (0.01)	857	0.88 (0.02)	133	0.88 (0.02)	182	0.88 (0.02)	**0.032**
MEF (FEF 25–75%)	1319	2.90 (0.29)	147	2.90 (0.30)	857	2.92 (0.29)	133	2.88 (0.29)	182	2.86 (0.30)	0.132
**Z-Scores**											
FEV_1_ (L)	1319	0.53 (0.99)	147	0.45 (0.97)	857	0.60 (0.97)	133	0.52 (1.00)	182	0.31 (1.08)	**0.002**
FVC (L)	1319	0.83 (1.10)	147	0.84 (1.13)	857	0.83 (1.10)	133	0.82 (1.05)	182	0.81 (1.17)	0.991
FEV_1_/FVC ratio	1319	−0.39 (1.10)	147	−0.57 (1.00)	857	−0.29 (1.10)	133	−0.41 (1.08)	182	−0.71 (1.15)	**< 0.001**
MEF (FEF 25–75%)	1319	−0.06 (1.06)	147	−0.28 (0.94)	857	0.05 (1.03)	133	−0.13 (1.13)	182	−0.37 (1.15)	**< 0.001**

*Note*: One way ANOVA mean comparison tests were performed to define the significance of the mean differences across the clusters; *Abbreviations*: *FEF* forced expiratory flow, *FEV*_1_ forced expiratory volume in 1 s, *FVC* forced vital capacity, *MEF* mid expiratory flow (FEF25–75%)

### Cluster 1: had asthma – currently healthy

This group consisted of children who suffered from early childhood asthma but currently had no asthma and accounted for 11.0% of the participants. Within the cluster, only 8.2% of the children had ongoing eczema. No children in this group reported suffering from any other comorbidities. The mean PedsQL scores of children in this cluster on the physical, psychosocial summary and total inventory were 84.5, 77.4 and 79.9, respectively. All these scores were very close to the scores of the *never asthmatic & healthy* cluster (Table 4). The average values of the lung function measures (FEV, FVC, FEV1/FVC ratio, MEF and their z-scores) among the children of this cluster were slightly higher than the *asthmatic unhealthy* cluster (Table 4).

### Cluster 2: never asthmatic & healthy

The *never asthmatic & healthy* cluster, the largest group of the children (64.9%), reported no incidence of asthma in their childhood or at present. Less than 5% of children within this cluster experienced wheezing, eczema, sleeping problems or reported at least one health condition; none suffered from a food allergy. Furthermore, only 7.1% of this group reported poor health. The average physical (84.0), psychosocial (76.7), and summary (79.3) PedsQL scores were higher than the early-onset asthmatic or allergic and asthmatic unhealthy clusters. The average scores of the lung functions measure (2.57, 2.99, 0.88 and 2.92 for FEV, FVC, FEV1/FVC ratio and MEF respectively) were also higher in this cluster compared to the early-onset asthmatic or allergic and asthmatic unhealthy clusters. Given that none of the children of this group ever had asthma or any ongoing condition associated with asthma, this was the healthiest cluster with respect to asthma, its related comorbidities, and the health outcome measures.

### Cluster 3: early-onset asthmatic or allergic

The *early-onset-asthmatic/allergic* cluster, comprising 10.7% of children, were suffering from multiple morbidities. Approximately 40% of this cluster were diagnosed with asthma in their early childhood, 15.8% were currently suffering from wheezing and 22.1% reported ongoing eczema. This group performed worse than the never asthmatic & healthy cluster with respect to PedsQL measures related to physical health. For inventory physical health summary, inventory psychosocial health summary and inventory total score, this cluster’s average scores were 81.5, 72.1 and 75.4, respectively, while the never asthmatic & healthy cluster’s scores were 84.0, 76.7 and 79.3 respectively (Table 4). The brief multi-dimensional student’s life satisfaction scale and overall life satisfaction of never asthmatic & healthy cluster were 83.0 and 81.0 which is better than early-onset-asthmatic/allergic cluster. The lung function average scores (FEV_1_, FVC, FEV_1_/FVC ratio, MEF and their z-scores) of this cluster were lower than those of the never asthmatic & healthy cluster (Table 4).

### Cluster 4: asthmatic unhealthy

Among the sample, 13.4% of children were classified as being in the asthmatic unhealthy cluster. Every child in this cluster was currently taking medication for asthma and all were diagnosed with asthma in their early childhood. Moreover, 38.7% had an illness with wheezing, just over one in five had either atopic eczema or reported not having excellent or very good health, and more than one in ten of them reported a sleeping disorder due to breathing or snoring problems. The average physical (79.4) and psychosocial (73.1) summary scores and the inventory total score (75.3) of this group were lower than the had asthma – currently healthy and never asthmatic & healthy groups. However, these scores were very close to the average scores of the children of the early-onset asthmatic or allergic cluster (Table 4). The average lung function values (2.46, 2.96, 0.88 and 2.86 for FEV_1_, FVC, FEV_1_/FVC ratio and MEF, respectively) of this cluster were lower than those of all other clusters (Table 4).

### Lung functions

**Fig. 2:**
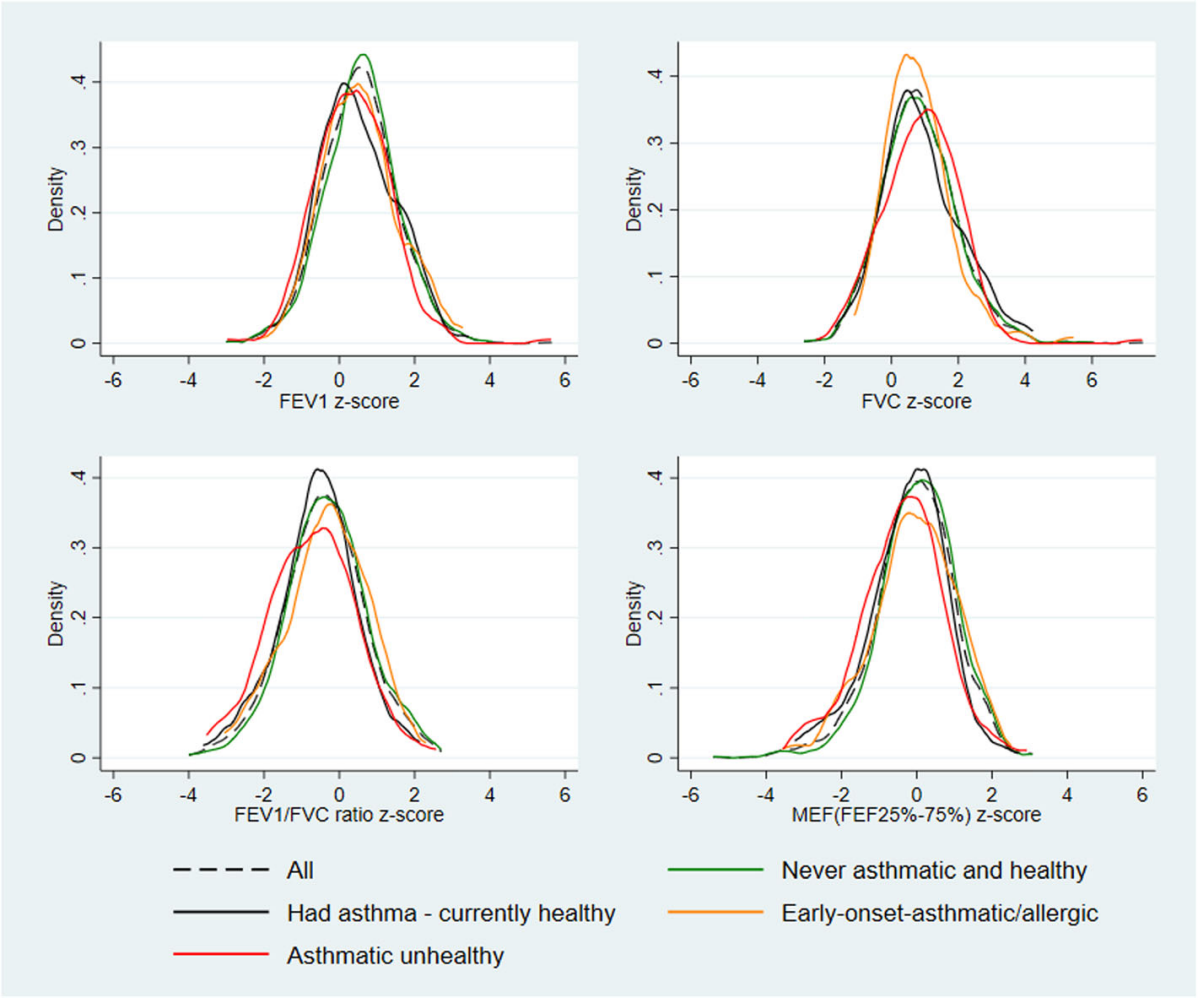
Lung function distribution and density plots for children by clusters. Abbreviations: FEF, forced expiratory flow; FEV_1_, forced expiratory volume in 1 s; FVC, forced vital capacity; MEF, mid expiratory flow (FEF25–75%)

**Fig. 3:**
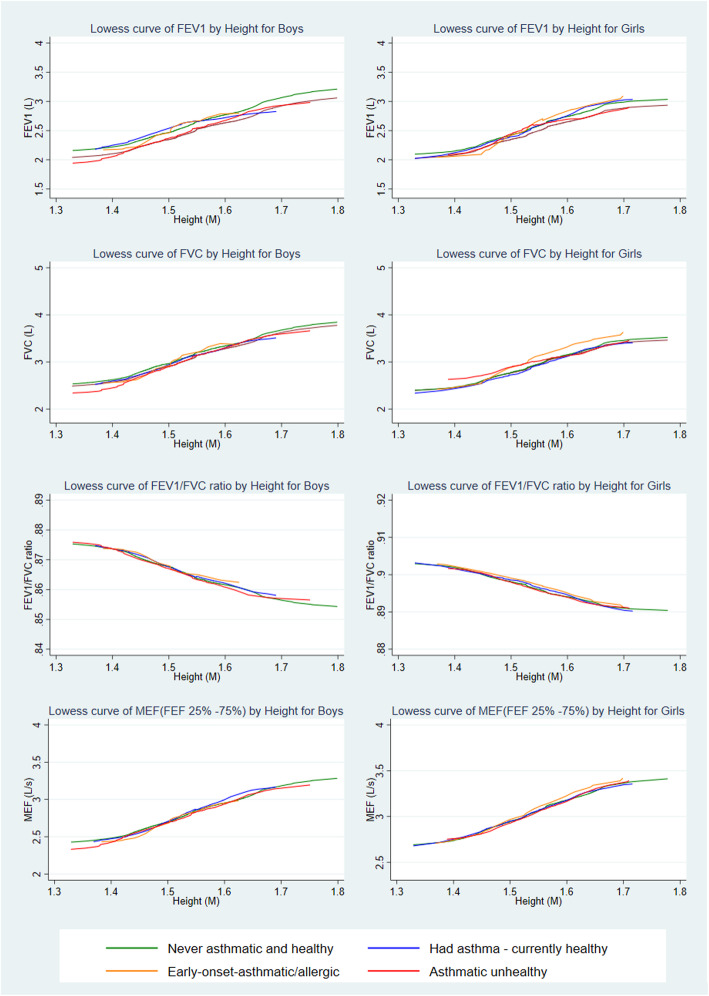
Lowess curve against height for the lung function of boys and girls by clusters. Abbreviations: FEF, forced expiratory flow; FEV_1_, forced expiratory volume in 1 s; FVC, forced vital capacity; MEF, mid expiratory flow (FEF 25–75%)

The distributions of the four different lung function measures (z-score of FEV, FVC, FEV1/FVC ratio and MEF) for the children of the full sample and each of the clusters are shown in Fig. 2. The asthmatic unhealthy cluster, followed by the early-onset asthmatic or allergic cluster, shows lower peaks compared to the never asthmatic & healthy cluster; all are fairly normally distributed. Figure 3 shows the LOWESS curve of all lung function measures segregated by sex against the children’s height for each of the clusters. These visual graphs clearly show that children in the asthmatic unhealthy cluster had lower spirometry results compared to all other clusters. In all clusters, shorter children had poorer lung function. In addition, the section C of Table 4 shows the mean, standard deviation (SD) and z-scores of FEV_1_, FVC, FEV_1_/FVC ratio and MEF measures among children by cluster.

Table 5 shows the prevalence of lung function impairments. The prevalence of decreased ventilator capacity (FEV_1_ less than the lower limit of normal (LLN)) was found among 8.86 and 5.25% of children in the asthmatic unhealthy (cluster 4) and had asthma – currently healthy (cluster 1) clusters, respectively. These values were 6.14 and 2.53 percentage points higher than those of the never asthmatic & healthy cluster (cluster 2). Similarly, more children in the asthmatic unhealthy cluster were in the critical zones (LLN < -2 z-value or LLN < 1.645 z-value) of FEV_1_ compared to other clusters (Table 5).

**Table 5:** Prevalence of decreased ventilator capacity, possible restrictive pattern, and obstruction as per the raw measures and Z-score (for both 2.5th and 5th percentile limit) of Health CheckPoint Survey, stratified by cluster and sex (in percent within the cluster)

	Cluster 1			Cluster 2			Cluster 3			Cluster 4		
	All (%)	Male (%)	Female(%)	All (%)	Male (%)	Female(%)	All (%)	Male (%)	Female(%)	All (%)	Male (%)	Female(%)
**Raw**												
Decreased ventilator capacity FEV_1_ (L) < LLN (L)	5.25	8.30	0.83	2.72	2.25	3.17	3.51	3.96	3.03	8.86	12.36	4.65
Possible restrictive pattern FVC (L) < LLN(L)	1.24	1.33	1.11	1.22	1.19	1.25	1.90	0	3.93	1.21	1.84	0.46
Obstruction FEV_1_/FVC ratio < LLN (80%)	0	0	0	0	0	0	0	0	0	0	0	0
Small Airway Impairment MEF (FEF 25–75%) < LLN (L/s)	18.65	21.14	15.04	11.46	11.32	11.60	14.84	10.94	19.03	26.10	32.16	18.80
**Z-score**												
***LLN for 2.5th percentile***												
FEV_1_ < -2 z score	4.93	7.75	0.83	0.67	0.64	0.70	1.28	2.48	0	5.08	8.79	0.61
FVC < −2 z score	0.45	0	1.11	0.35	0.28	0.43	1.07	0	2.21	1.01	1.84	0
FEV_1_/FVC ratio < −2 z score	16.95	19.79	12.83	10.19	10.18	10.20	10.57	11.72	9.33	21.65	27.32	14.81
MEF (FEF 25–75%) < − 2 z score	12.00	14.68	8.12	6.32	6.03	6.59	10.32	9.30	11.41	18.62	24.05	12.08
***LLN for 5th percentile***												
FEV_1_ < -1.645 z score	5.25	8.30	0.83	2.72	2.25	3.17	3.51	3.96	3.03	8.86	12.36	4.65
FVC < −1.645 z score	1.24	1.33	1.11	1.22	1.19	1.25	1.90	0	3.93	1.21	1.84	0.46
FEV_1_/FVC ratio < −1.645 z score	22.34	24.27	19.55	17.63	17.38	17.87	18.24	16.63	19.96	33.57	38.35	27.81
MEF (FEF 25–75%) < − 1.645 z score	18.65	21.14	15.04	11.46	11.32	11.60	14.84	10.94	19.03	26.10	32.16	18.80

*Abbreviations*: *FEF* forced expiratory flow, *FEV*_1_ forced expiratory volume in 1 s, *FVC* forced vital capacity, *MEF* mid expiratory flow (FEF25–75%), *LLN* lower limit to normal

The prevalence of possible restrictive patterns (FVC < LLN) was negligible among the children across all the clusters. Airway obstruction was not present among the children of all clusters when we considered 80% of the predicted value for the FEV_1_/FVC ratio to be the lower limit of normal. However, obstruction was present among the children across the clusters if we considered the z-score of − 2 for the FEV_1_/FVC ratio to be the lower limit of normal. Then, the highest prevalence (21.65%) was observed among the children of the asthmatic unhealthy cluster and the lowest (10.19%) was in the never asthmatic & healthy cluster (Table 5).

The lack of flow rate results between 25 and 75% vital capacity (MEF < LLN L/s) indicated small airway impairment among the children in all the clusters. The lowest prevalence (11.46%) was among the children of the never asthmatic & healthy cluster and the highest (26.10%) was among the children of the asthmatic unhealthy cluster. However, a lower prevalence was observed if an MEF z-score of − 2 (2.5th percentile) was considered to be the lower limit of normal. Then, the impairment ranged from 6.32 to 18.62% among the clusters.

### Regression results

Results from a multinomial regression analysis revealed that children from mothers who experienced asthma during their pregnancy were 3.31 times (OR 3.31, 95% CI: 2.07–5.30) more likely to fall into the asthmatic unhealthy cluster, compared to the children of the never asthmatic & healthy cluster (Table 6). The study also found that if the mothers had any of the medical conditions diagnosed during the year of childbirth, their children were 1.72 times (OR: 1.72, 95% CI: 1.23–2.42) more likely to belong to the asthmatic unhealthy group. The findings also confirmed that the children from mothers who had asthma during pregnancy were 2.5 times (OR: 2.50, 95% CI: 1.42–4.39) more likely to experience early childhood asthma, though they might have been cured before the age of 12–13 years, compared to the children from mothers who did not have asthma during pregnancy.

**Table 6:** Multinomial logistic regression of class memberships on covariates of health risk exposures of mothers during pregnancy or in the year of childbirth^a^

health risk exposures of mothers	Had asthma – currently healthy		Early-onset-asthmatic/allergic		Asthmatic unhealthy	
	OR (95% CI)	*p*-value	OR (95% CI)	*p*-value	OR (95% CI)	*p*-value
Mother had asthma during pregnancy						
*No (ref.)*						
*Yes*	**2.50 (1.42–4.39)**	**0.002**	1.7 (0.91–3.18)	0.093	**3.31 (2.07–5.30)**	**< 0.001**
Had any medical condition in the year of childbirth						
*No (ref.)*						
*Yes*	1.03 (0.69–1.54)	0.872	1.23 (0.84–1.8)	0.297	**1.72 (1.23–2.42)**	**0.002**
General health status of mother						
*Excellent/Very good health (ref.)*						
*Good health*	0.93 (0.63–1.38)	0.73	1.45 (1–2.09)	0.051	1.26 (0.88–1.8)	0.208
*Fair/Poor health*	1.53 (0.81–2.89)	0.190	1.22 (0.63–2.36)	0.561	1.7 (0.96–3.01)	0.069
Obesity status of mothers before pregnancy						
*Healthy weight (ref.)*						
*Underweight*	0.91 (0.5–1.65)	0.750	1.21 (0.69–2.12)	0.495	0.96 (0.55–1.69)	0.885
*Overweight*	1.41 (0.94–2.1)	0.094	1.09 (0.72–1.66)	0.688	1.23 (0.83–1.81)	0.301
*Obese*	1.1 (0.66–1.82)	0.724	1.07 (0.65–1.75)	0.803	1.19 (0.75–1.89)	0.462
Birth Weight of children						
*Healthy weight (ref.)*						
*Low birth weight (< 2500 g)*	1.42 (0.61–3.3)	0.410	0.86 (0.34–2.19)	0.754	1.91 (0.87–4.18)	0.106
*High birth weight (> = 4000 g)*	1.41 (0.92–2.16)	0.110	1.23 (0.79–1.94)	0.361	0.87 (0.55–1.38)	0.553
Gestational Age of birth						
*On time birth (ref.)*						
*Pre-term birth (< 37 weeks)*	1.22 (0.56–2.65)	0.613	1.29 (0.58–2.87)	0.534	0.87 (0.4–1.88)	0.715
*Late birth (> = 42 weeks)*	0.73 (0.3–1.77)	0.493	1.07 (0.5–2.27)	0.859	1.34 (0.69–2.59)	0.385
Frequency of smoking in 1st Trimester						
*No smoking (ref.)*						
*Occasional / < 10 cigarettes per day*	0.35 (0.14–0.93)	0.035	0.87 (0.44–1.72)	0.699	1.28 (0.72–2.27)	0.403
*10+ cigarettes per day*	1.04 (0.43–2.57)	0.924	1.33 (0.58–3.04)	0.496	1.34 (0.63–2.86)	0.449

^a^*Note*: The Multinomial logistic regression model has been constructed using *never asthmatic & healthy cluster* as reference category. Further, for each of the independent variables, the reference category (ref.) has been mentioned at the beginning of the categorical values. The regression model has been controlled with mothers’ other health related and socio-economic variables listed as follows: breastfeeding, type of birth delivery, immunisation status of children, gender of the child, education and marital status of mother, family income, language spoken at home and remoteness of the residence

## Discussion

This study investigated a birth cohort of Australian children (*n* = 1777) aged 12–13 years and applied LCA to identify four statistically distinct clusters based on the prevalence of asthma and related comorbidities. The four clusters were defined as (i) had asthma – currently healthy, (ii) never asthmatic & healthy, (iii) early-onset asthmatic or allergic, and (iv) asthmatic unhealthy. The clusters’ characteristics were described and compared based on the specific health outcomes as measured by spirometry, PedsQL and general wellbeing. Findings of the regression modelling revealed that children whose mothers had asthma during pregnancy were more likely to be in the asthmatic unhealthy cluster than children of non-asthmatic mothers.

This study found that the mean scores of the FEV1, FVC, and MEF measures of the children of the never asthmatic & healthy cluster were higher than the values of the healthy reference population of Hibbert et al. [28]. This study also found that most of these values for the asthmatic unhealthy cluster children were lower than the values of the healthy reference population of Hibbert et al. [28]. These study findings support the earlier literature’s assessment that clinical asthma is correlated with lower airway function, with variations depending upon the severity of the asthma [27].

One-fifth of the asthmatic cluster children showed signs of airway obstruction when an FEV1/FVC z-score < − 2 was considered. This measure indicates airway size relative to lung volume, which was lower among asthmatic unhealthy children [26]. This measure may indicate the risk of several health issues among the children of this group, including dysanaptic lung growth and airway obstruction [26, 29, 30]. However, this ratio may be lower in a portion of the children due to differences in measurement technique and equipment or the influence of gender-specific pubertal status [26].

The lack of performance in the measures of mid-expiratory flow (MEF, FEF 25–75% < LLN L/s) revealed a common prevalence of small airway impairment among the children of all four clusters to varying extents. The highest prevalence of this small airway impairment was observed in the asthmatic unhealthy cluster, where 1 in 4 children were affected. A study undertaken by Marseglia et al. revealed that small airway impairment or disease was present among subjects who were affected with early allergic or inflammatory symptoms with allergic disease and no asthma [31]. A portion of children from all the clusters of this study population were affected by small airway disease, perhaps due to atopic allergy or food allergy across all the clusters. A Western Australian study by Palmer et al. [32] also found that the presence of asthma lowered spirometry performance. Xu et al. revealed significant impairment of lung function in the families of both children with asthma and healthy non-asthmatic children [33]. The predominance of obstruction, decreased ventilator capacity and possible restrictive pattern in the *asthmatic unhealthy* cluster revealed by the four measures of pulmonary functions support the findings of Palmer et al., Xu et al. and Weatherall et al. [12, 32, 33].

Our regression analyses showed that ‘membership’ in the *asthmatic unhealthy* group was significantly associated with the incidence of maternal asthma during pregnancy. These findings are consistent with previous research that found that maternal asthma was significantly associated with the increased prevalence of asthma in children [32]. The incidence of maternal medical conditions during the year of pregnancy also significantly increased the likelihood of a child’s classification in the asthmatic unhealthy group. The severity of children’s health conditions in the asthmatic unhealthy group was aggravated to a great extent by the comorbidities of wheezing, eczema, snoring or breathing problems, or food allergies. Analogous to the findings of this study, Martel et al. found that the severity of mothers’ asthma, lack of asthma control and comorbidity with any medical conditions were all associated with increased incidences of recurring asthma in their children, along with the possible comorbidities of wheezing, eczema, snoring or breathing problems and food allergies [17].

In contrast with other studies [34, 35], our study found no evidence that maternal smoking during pregnancy increased the probability of a child being included in the asthmatic unhealthy cluster. One reason might be that this study investigated the association with the cluster memberships, rather than with the whole sample of children affected by asthma morbidity in early childhood or at present. Our findings warrant further research, as previous studies have shown evidence of an association between maternal smoking and childhood asthma [15, 34–37]. Future studies may consider the significant decline in Australian smoking rates [38], which might contribute to the decrease in this adverse health outcome at the national population level.

The study’s strengths included its utilisation of cluster analysis instead of characterisation of isolated individual morbidities and its examination of co-factors related to asthma to investigate the differences in health status between asthma morbidity clusters. This study utilized Australia’s nationally representative sample which would help understand the cluster identifications for the adolescents at national level. A limitation included its dependence on self-reported data for general health, wellbeing and PedsQL measures. There were some missing data for the lung function measurements, which may modify the interpretation of the analyses.

## Conclusion

This study supports the necessity to consider multiple morbidity factors related to asthma when classifying individuals and identifying high-risk asthma groups. Our analyses identified four main clusters of children, based on their experiences of asthma and related morbidities and their association with maternal health during pregnancy. The most vulnerable group was the asthmatic unhealthy cluster and children whose mothers had asthma during pregnancy were threefold more likely to be in this cluster. This study suggests that an improved classification strategy helps to identify the most vulnerable group among the children with asthma and related morbidities.

## Data Availability

There are some restrictions on the use of this data and the data application’s approval is subject to a signed confidentiality deed. Those interested in accessing this data should contact the Longitudinal Study of Australian Children Dataverse of NCLD, Australian Government Department of Social Services over email or lodge an online application from the following web link: https://growingupinaustralia.gov.au/data-and-documentation/ accessing-lsac-data.
